# Needle artifact reduction during interventional CT procedures using a silver filter

**DOI:** 10.1186/s42490-024-00076-y

**Published:** 2024-03-11

**Authors:** Carlos A. Reynoso-Mejia, Jonathan Troville, Martin G. Wagner, Bernice Hoppel, Fred T. Lee, Timothy P. Szczykutowicz

**Affiliations:** 1 Department of Radiology, University of Wisconsin–Madison, Madison, WI 53705 USA; 2 Department of Medical Physics, University of Wisconsin–Madison, Madison, WI 53705 USA; 3 Canon Medical Systems USA, Irvine, CA 92618 USA; 4 Department of Urology, University of Wisconsin–Madison, Madison, WI 53705 USA; 5 Department of Biomedical Engineering, University of Wisconsin–Madison, Madison, WI 53705 USA

**Keywords:** Interventional CT, CT artifact, CT filter

## Abstract

**Background:**

MAR algorithms have not been productized in interventional imaging because they are too time-consuming. Application of a beam hardening filter can mitigate metal artifacts and doesn’t increase computational burden. We evaluate the ability to reduce metal artifacts of a 0.5 mm silver (Ag) additional filter in a Multidetector Computed Tomography (MDCT) scanner during CT-guided biopsy procedures.

**Methods:**

A biopsy needle was positioned inside the lung field of an anthropomorphic phantom (Lungman, Kyoto Kagaku, Kyoto, Japan). CT acquisitions were performed with beam energies of 100 kV, 120 kV, 135 kV, and 120 kV with the Ag filter and reconstructed using a filtered back projection algorithm. For each measurement, the CTDIvol was kept constant at 1 mGy. Quantitative profiles placed in three regions of the artifact (needle, needle tip, and trajectory artifacts) were used to obtain metrics (FWHM, FWTM, width at − 100 HU, and absolute error in HU) to evaluate the blooming artifact, artifact width, change in CT number, and artifact range. An image quality analysis was carried out through image noise measurement. A one-way analysis of variance (ANOVA) test was used to find significant differences between the conventional CT beam energies and the Ag filtered 120 kV beam.

**Results:**

The 120 kV-Ag is shown to have the shortest range of artifacts compared to the other beam energies. For needle tip and trajectory artifacts, a significant reduction of − 53.6% (*p* < 0.001) and − 48.7% (*p* < 0.001) in the drop of the CT number was found, respectively, in comparison with the reference beam of 120 kV as well as a significant decrease of up to − 34.7% in the artifact width (width at − 100 HU, *p* < 0.001). Also, a significant reduction in the blooming artifact of − 14.2% (FWHM, p < 0.001) and − 53.3% (FWTM, p < 0.001) was found in the needle artifact. No significant changes (*p* > 0.05) in image noise between the conventional energies and the 120 kV-Ag were found.

**Conclusions:**

A 0.5 mm Ag additional MDCT filter demonstrated consistent metal artifact reduction generated by the biopsy needle. This reduction may lead to a better depiction of the target and surrounding structures while maintaining image quality.

## Background

**Fig. 1:**
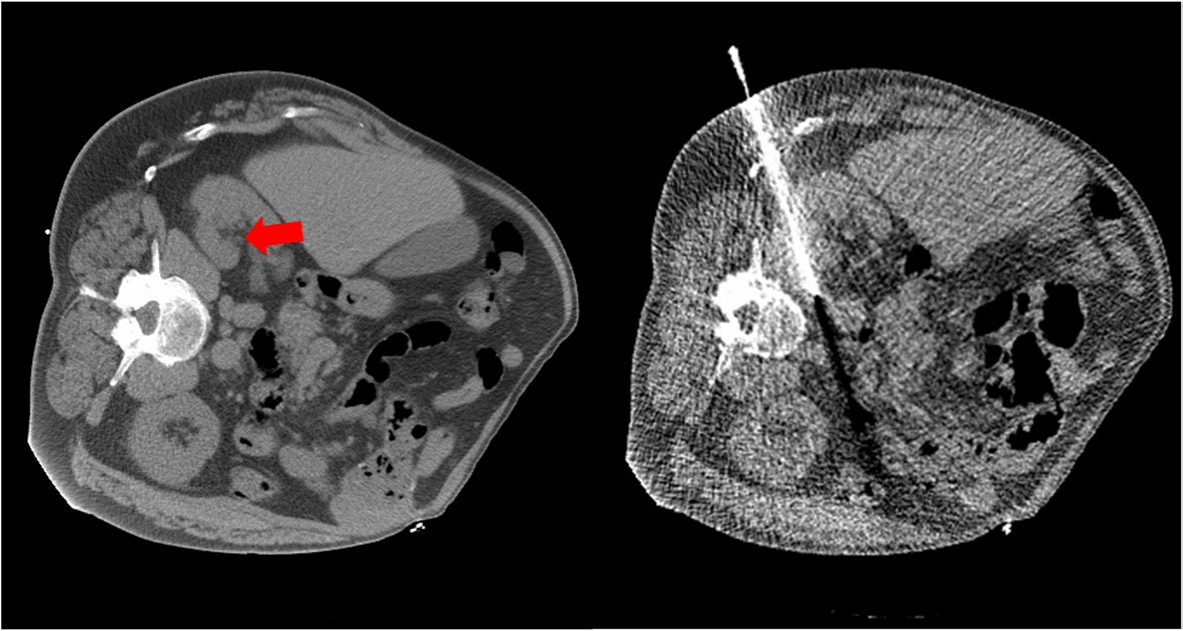
Metal artifacts from biopsy needles can obscure targets. In this example, a planning CT is shown on the left, and an intraprocedural CT fluoroscopy image on the right. The target is a 1.8 cm lymph node which is easily visualized on the left image (red arrow) but completely obscured by an artifact on the right. Interventional device artifacts commonly manifest themselves in this way, hindering both visualization of important anatomy that will be traversed by the needle, and localization of the target

Minimally invasive CT-guided percutaneous procedures require an adequate depiction of the device tip and the target lesion to achieve a successful result. Metal artifacts generated by metal trocars such as introducer needles may interfere with the visualization of the target region as well as increase uncertainty during needle placement [1]. Stattaus et al. [2] found that the rate of insufficiently visualized targets increased from 10.5% on prebiopsy images to 44.7% with the needle within the lesion due to needle artifact. Techniques such as microwave ablation also require precise positioning between the ablation antenna and the lesion to achieve successful therapy [1, 3] and suffer from similar metal artifacts. Figure 1 depicts an example of a typical metal induced artifact during an interventional CT procedure.

Different artifacts are created due to the metallic object within the field of view. The needle will harden the x-ray beam due to the increased absorption of the low-energy photons in the metal, causing beam-hardening artifacts. The high attenuation of the photons in the metal also causes photon starvation, creating streak artifacts with artificial hypointense regions and bright streaks due to insufficient photons reaching the detector [4]. Additionally, physical effects such as scattered radiation, nonlinear partial volume, and a significant increase in noise cause image degradation [5]. Figure 1 shows a CT fluoroscopy image of a biopsy in which image degradation due to artifacts generated by the needle obscures the target lesion.

Several strategies have been proposed to reduce metallic artifacts in CT images. McWilliams et al. [6] found that streak artifacts can be reduced by removing the central stylet of the needle guide and using smaller-gauge guide needles. Recently, metal artifact reduction (MAR) algorithms have become available for CT imaging, mainly designed to reduce metal artifacts generated by metal implants. While these algorithms use proprietary information, they are sinogram-based and work through identifying, deleting, and replacing corrupted raw data [7–10]. Thereby, MAR algorithms may modify the non-artifactual image data while processing the artifact area, which could affect the perceived location of the target region [11, 12]. Additionally, many vendors do not have MAR algorithms in interventional CT imaging modes. In this study, we explore a method for reducing metal artifacts that does not require changing the stylet or using MAR algorithms. We investigate the use of a 0.5 mm Ag (i.e., Silver) additional filter in an MDCT scanner that hardens the x-ray beam. We specifically evaluate the Ag filter’s ability to reduce metal artifacts for interventional CT procedures.

## Materials and method

### Image protocol

An anthropomorphic phantom (Lungman, Kyoto Kagaku, Kyoto Japan) [13] simulating a patient was scanned with a biopsy needle, and its sheath (Chiba Biopsy Needle 18G/15 cm, Cook Medical) aligned in the axial plane inside the lung field. Images of the phantom and needle were acquired using an MDCT scanner (Aquilion One Prism Edition, Canon Medical USA) which incorporates an optional 0.5 mm additional Ag filter (SilverBeam™). Three images of the same region were acquired for beam energies of 100 kV, 120 kV, 135 kV with no additional Ag filter, and 120 kV with the additional Ag filter. The filter has been previously characterized and was shown to increase the effective beam energy at 120 kV from 66.2 keV to 80.9 keV (22%) [14]. This is similar to the effect of using a tin (Sn) filter which has been well documented in the literature. For example, The Sn filter increases the mean weighted energy from 58.7 keV to 76.0 keV for 100 kV (29%) and from 72.1 to 98.6 keV for 150 kV (37%) [15]. The vendor only allowed the use of the Ag filter at 120 kV. For each measurement, the CTDIvol (i.e., a surrogate for patient dose) was kept constant at 1 mGy which is a typical interventional CT dose at our institution. Tube current modulation was not used for image acquisition. Images were reconstructed using a filtered back projection algorithm with the FC18 kernel, a slice thickness of 2 mm, and a 512 × 512 matrix.

### Quantitative analysis

**Fig. 2:**
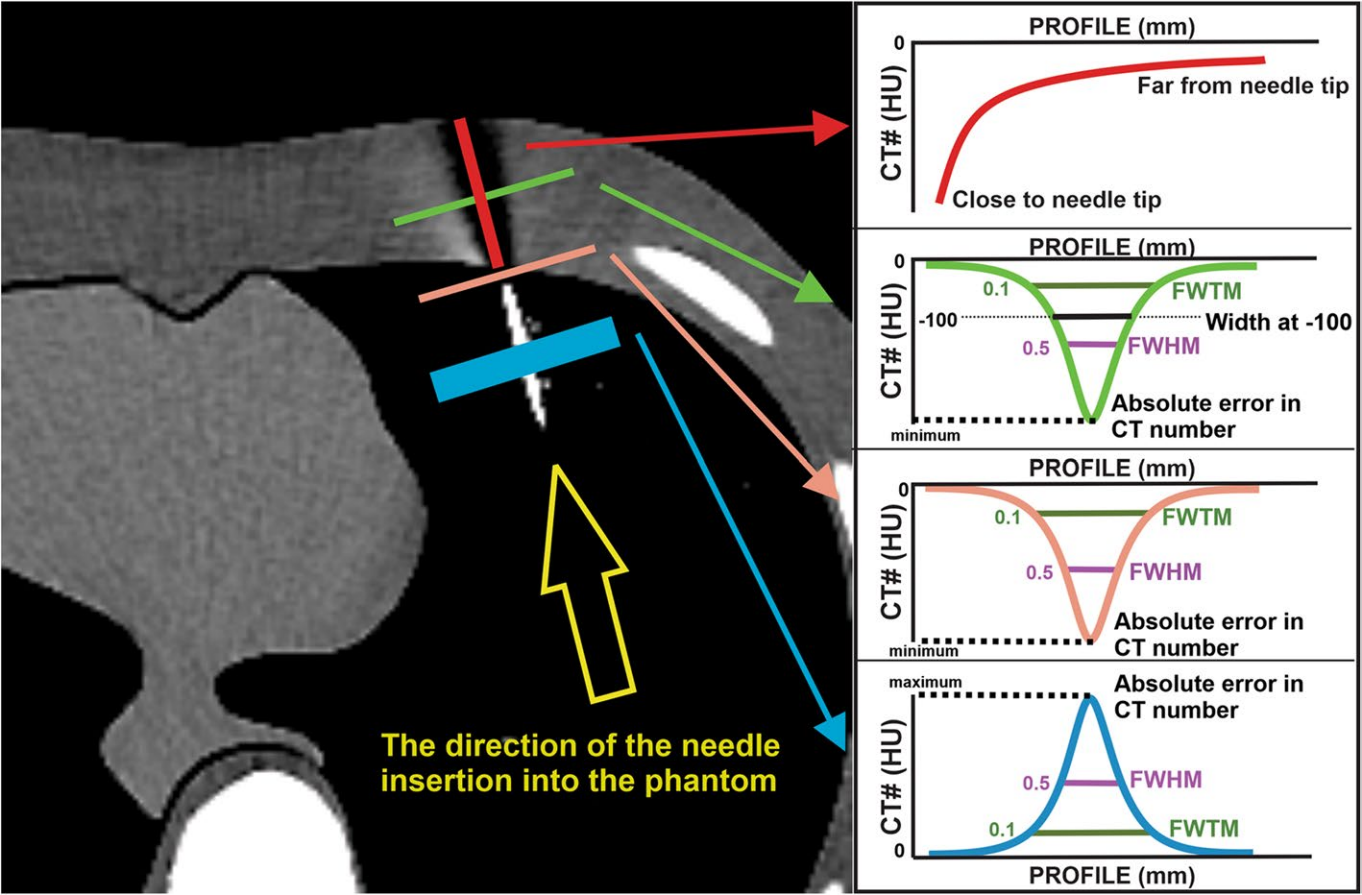
Shown are the positions in the CT image where the profiles were drawn to obtain the values of FWHM, FWTM, and the absolute error in HU used to quantify the artifacts generated from the biopsy needle. FWHM = full width half maximum, and FWTM = full width at one-tenth of maximum (FWTM). The needle was inserted from the back of the right lung until the needle tip reached the region of tissue at chest level

For the assessment of artifacts, quantitative profiles were drawn on three locations surrounding the needle which are important for physician device control. Figure 2 shows the positions of the profiles. The profiles represent changes in the Hounsfield Unit (HU) from non-artifactual background regions. Three profiles were placed perpendicular to the needle trajectory: 1) on the needle (to evaluate the blooming artifact of the needle). 2) needle tip artifact (the darkest region, immediately in front of the needle tip), and 3) trajectory artifact (i.e., the width of the artifact in the region in front of the needle). One profile was placed along the needle to measure the range of the artifact along the trajectory of the needle.

Each profile was sampled by dividing the voxel size of 0.976 mm by four (0.244 mm) using cubic spline interpolation. To estimate the width of the artifacts, the full width at half maximum (FWHM) and full width at one-tenth of maximum (FWTM) of the profiles were calculated. Additionally, the width of the profiles at a Hounsfield unit difference of − 100 HU was also measured. The absolute error (AE) corresponding to the maximum change in the CT number due to the artifact for each profile was also obtained. Figure 2 shows how the quantitative values (FWHM, FWTM, and AE) were obtained from the profiles.

### Image noise

**Fig. 3:**
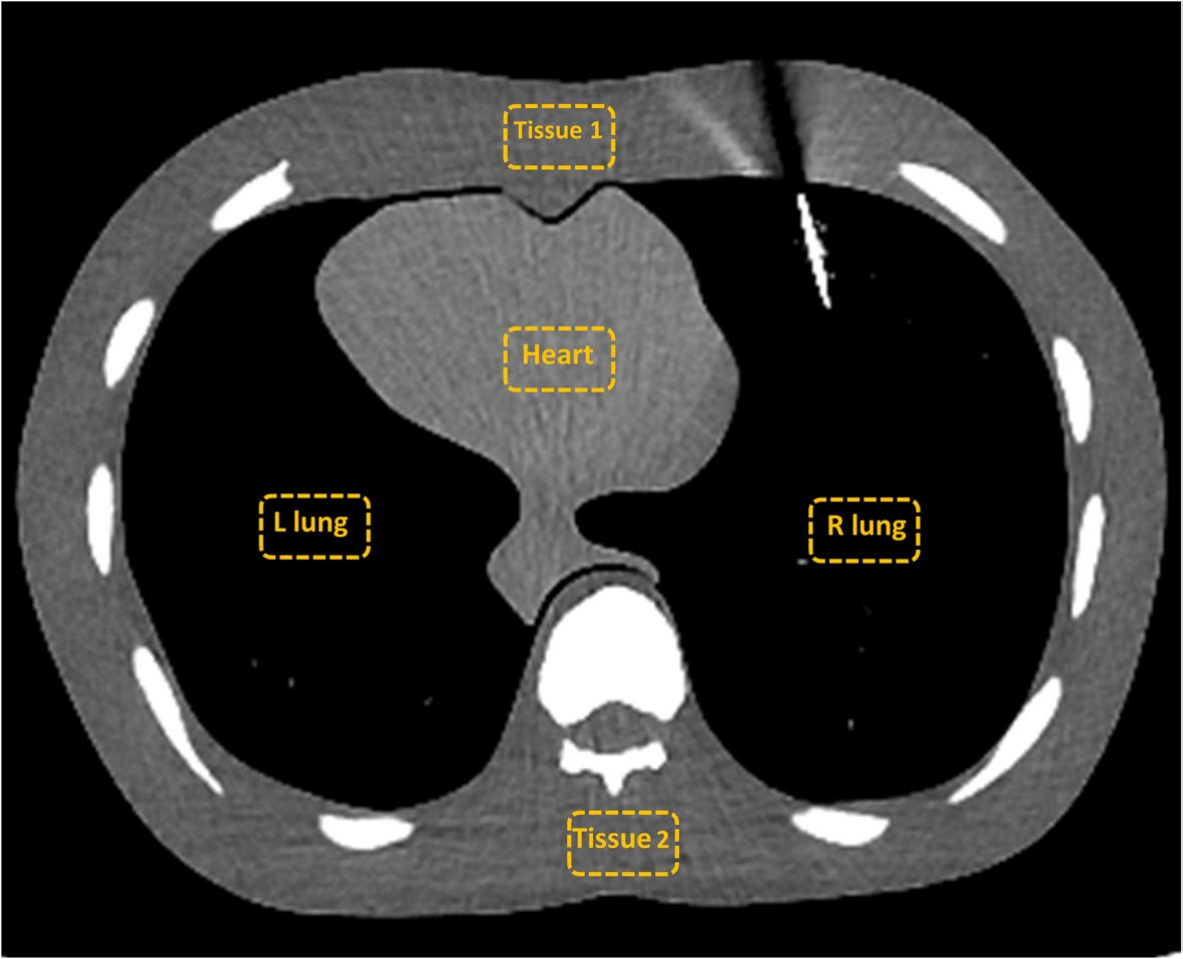
CT image showing the ROIs positions (yellow rectangles) used to measure the image noise

Image quality assessment was performed through quantitative comparisons of the image noise (i.e., pixel standard deviation from a uniform region) in five rectangular regions of interest (ROI) of approximately 200 mm^2^. ROIs were placed outside the artifact region on the heart, left lung, right lung, tissue 1, and tissue 2. Figure 3 shows the ROIs’ position on the CT image. The same ROI positions were applied to all beam energies. The image noise (standard deviation of the pixel HU value) was obtained in each ROI.

### Statistical analysis

All measurements were repeated three times. A one-way analysis of variance (ANOVA) was used to find the significant differences among the FWHM, FWTM, width for − 100 HU, and absolute error for 100 kV, 135 kV, and 120 kV-Ag with the reference beam of 120 kV. *p* < 0.05 was the criterion for statistical significance. To compare the image noise in the four beam energies among each beam energy, the Friedman test was used. All data analyses were performed using MATLAB (MathWorks vR2021a).

## Results

**Fig. 4:**
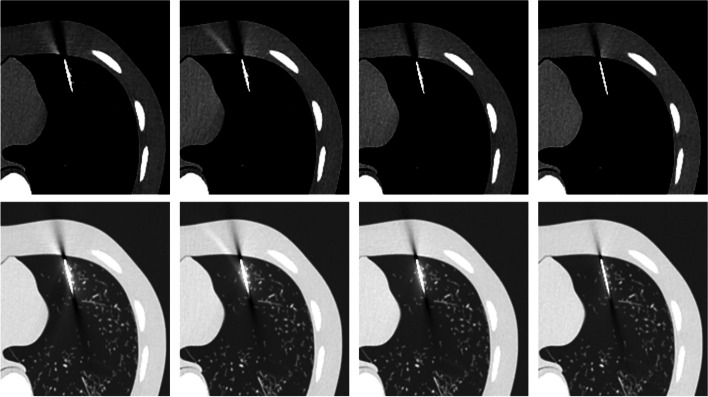
Axial CT images of the Lungman phantom with the needle inserted into the lung. The top row shows CT images reconstructed with a soft tissue window (ww/wl of 400/50) and the bottom row with a window for lung (ww/wl of 1700/− 700). Beam energy is indicated from left to right: 100 kV, 120 kV, 135 kV, and 120 kV with Ag additional filter

Our results show a visible decrease in metal artifacts generated by the biopsy needle when the silver filter is incorporated compared to beam energies of 100, 120, and 135 kV. A visible decrease in the streak artifacts and the needle’s blurring effect is observed for the beam energy of 120 kV with the Ag additional filter. This decrease is more clearly seen in the lung window. Figure 4. It also shows that the artifacts decrease as the beam is hardened.

**Fig. 5:**
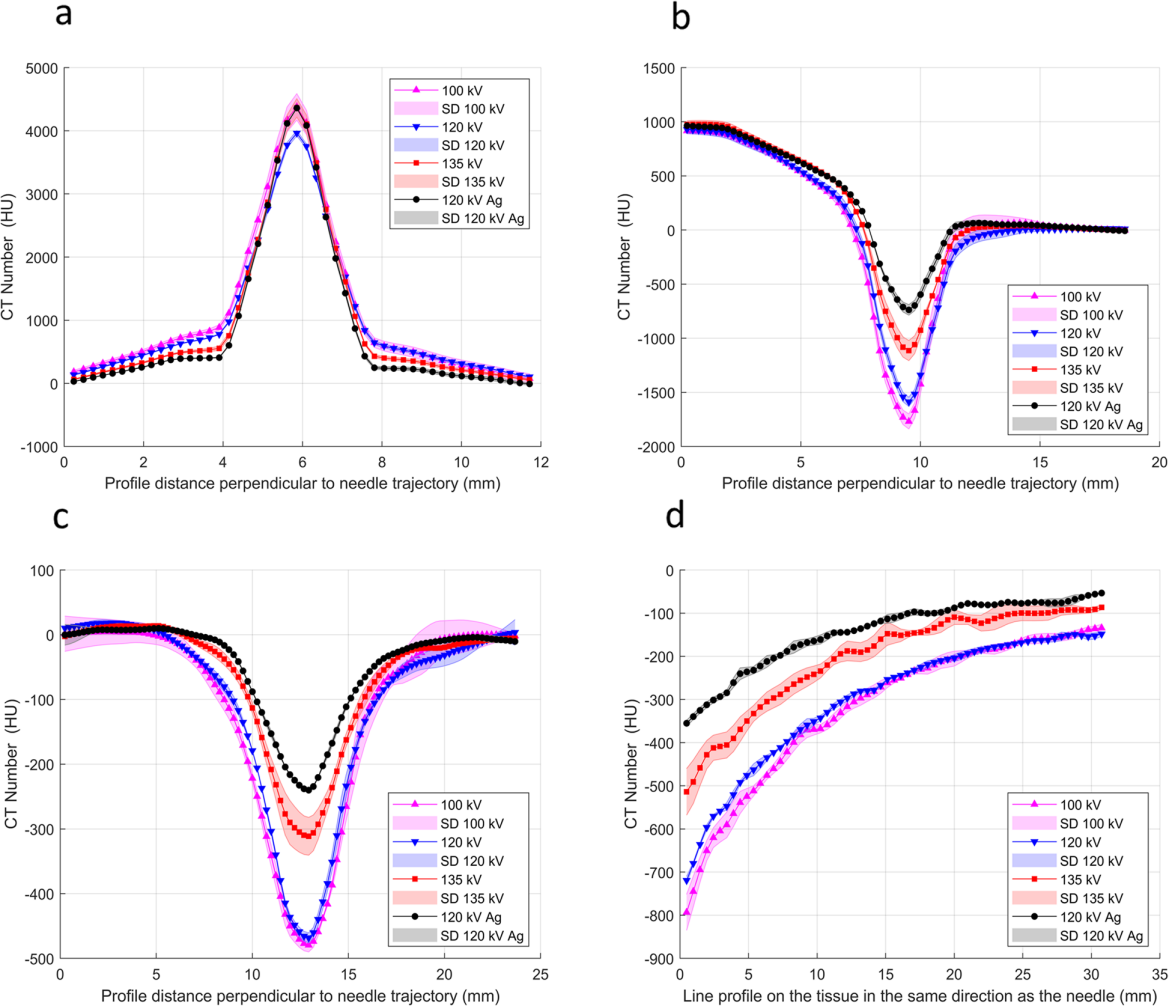
Profiles of the artifacts for the beam energies of 100 kV, 120 kV, 135 kV, and 120 kV-Ag filter. Profiles represent the Hounsfield unit change with the distance passing through the artifact. **a** profile on the needle (i.e., the yellow region in Fig. 2), **b** profiles in the needle tip artifact (i.e., the green region in Fig. 2), **c** profiles in the needle trajectory path artifact (i.e., the blue region in Fig. 2), and **d** profiles in the directions of the needle path on needle trajectory artifact (i.e., the red region in Fig. 2). The shaded regions depict standard deviations in profiles

Artifact profiles are shown in Fig. 5. The profiles on the needle (Fig. 5a) show an increase in the HU due to the needle that exceeds 4000 HU (AE > 4000 HU). The smallest blooming profile width was obtained for the 120 kV-Ag additional filter. The profiles on the needle tip artifact (Fig. 5b) show a decrease in HU due to the high attenuation of photons in the needle, reaching an absolute error of 1768 ± 69 HU at the 100 kV profile. The profile for 120 kV-Ag showed a relatively small decrease in HU (absolute error of 737 ± 48 HU) and the smallest width profile (FWHM of 2.08 ± 0.02 mm). Figure 5c shows the trajectory artifact profiles. A drop in HU is observed where the profiles pass through the artifact. However, the decrease in the HU is more moderate than at the needle tip. The maximum absolute error obtained was 479 ± 13 HU for the beam energy of 100 kV. The profile for the 120 kV-Ag showed the lowest absolute error (240 ± 79 HU) and the lowest width (FWHM of 4.25 ± 0.12 mm). Figure 5d shows the range profiles. The HU continuously decreased from the maximum value near the needle tip until they reach the zero value (background tissue). The profile for the 120 kV-Ag had the HU values closest to zero throughout the trajectory, indicating having the shortest range.

Table 1 shows the values of FWHM, FWTM, width at − 100 HU, and absolute error in the CT number for each beam energy. The table shows statistical *p*-values represent comparing metric scores for 100 kV, 135 kV, and 120 kV-Ag filter to the reference beam of 120 kV. Overall, the smallest FWHM values were obtained for the 120 kV-Ag. Compared the 120 kV-Ag with the reference beam of 120 kV, reductions of 0.16 ± 0.15 mm (− 3.6%), 0.36 ± 0.03 mm (− 14.8%), and 0.31 ± 0.04 mm (− 14.2%) were found for trajectory, needle tip, and needle artifacts, respectively. However, significant differences were obtained only for the needle artifact (2.19 ± 0.02 mm vs. 1.88 ± 0.04 mm; *p* < 0.05) and tip artifacts (2.44 ± 0.07 mm vs. 2.08 ± 0.02 mm; p < 0.05). The lowest FWTM values were also found for the 120 kV-Ag. Differences with respect to the reference beam of 120 kV were 2.25 ± 1.31 mm (− 19.9%), 0.94 ± 0.35 mm (− 23.2%), and 4.10 ± 0.17 mm (− 53.3%) for trajectory, needle tip, and needle artifacts, respectively. Significant differences were found for the needle artifact (7.69 ± 0.1 mm vs. 3.59 ± 0.009 mm; *p* < 0.05) and tip artifacts (4.05 ± 0.34 mm vs. 3.11 ± 0.08 mm; p < 0.05). The width at − 100 HU shows a significant decrease for the 120 kV-Ag with the reference beam of 120 kV (7.49 ± 0.23 mm vs. 4.89 ± 0.03 mm; p < 0.05), a difference of 2.60 ± 0.23 mm (34.7%). The AE measures also showed a significant decrease when the 120 kV-Ag was used in comparison with the reference beam of 120 kV for needle tip artifacts (1587 ± 42 HU vs. 737 ± 48 HU; p < 0.05) and trajectory artifacts (468 ± 8 HU vs. 240 ± 3 HU; p < 0.05) with reductions of 228 ± 8 HU (− 48.7%) and 850 ± 63 HU (− 53.6%), respectively. No significant differences in the absolute error were found for the needle artifact (*p* > 0.05). Table 1 position.

**Table 1:** The table shows the average values and their standard deviation for FWHM, FWTM, width at − 100 HU, and AE in CT number for the different beam energies. *p*-values represent comparing metric scores to the 120 kV case. Artifact regions are shown in Fig. 2

Metric	100 kV			120 kV			135 kV			120 kV- Ag		
	Trajectory artifact	Needle tip artifact	Needle artifact	Trajectory artifact	Needle tip artifact	Needle artifact	Trajectory artifact	Needle tip artifact	Needle artifact	Trajectory artifact	Needle tip artifact	Needle artifact
FWHM (mm) Difference % with 120 kV	**4.91 ± 0.15** **(*p*** **= 0.026)** **11.3**	2.37 ± 0.07 (*p* = 0.702) −2.9	2.17 ± 0.04 (*p* = 0.885) − 0.9	4.41 ± 0.09	2.44 ± 0.07	2.19 ± 0.02	4.51 ± 0.09 (*p* = 0.892) 2.3	2.31 ± 0.03 (*p* = 0.310) − 5.3	**1.98 ± 0.03** **(*p*** **= 0.003)** **− 9.6**	4.25 ± 0.12 (*p* = 0.677) − 3.6	**2.08 ± 0.02 (*p*** **= 0.007)** **− 14.8**	**1.88 ± 0.04 (*p*** **< 0.001)** **− 14.2**
FWTM (mm) Difference % with 120 kV	10.06 ± 0.59 (*p* = 0.500) − 10.8	3.81 ± 0.13 (*p* = 0.679) − 5.9	7.42 ± 0.73 (*p* = 0.937) − 3.5	11.28 ± 1.24	4.05 ± 0.34	7.69 ± 0.17	9.47 ± 0.29 (*p* = 0.296) − 16.0	3.53 ± 0.02 (*p* = 0.225) − 12.8	**5.36 ± 0.34 (*p*** **= 0.016)** **− 30.3**	9.03 ± 0.41 (*p* = 0.169) − 19.9	**3.11 ± 0.08 (*p*** **= 0.029)** − **23.2**	**3.59 ± 0.01 (p < 0.001)** − **53.3**
Width at −100 HU (mm) Difference % with 120 kV	7.85 ± 0.30 (*p* = 0.569) 4.8	n/a	n/a	7.49 ± 0.23	n/a	n/a	**5.97 ± 0.35 (*p*** **= 0.010)** **− 20.3**	n/a	n/a	**4.89 ± 0.03 (p < 0.001)** **− 34.7**	n/a	n/a
AE in CT number (HU) Difference % with 120 kV	479 ± 10 (*p* = 0.887) 2.4	1768 ± 69 (*p* = 0.130) 11.4	4379 ± 209 (*p* = 0.091) 10.5	468 ± 8	1587 ± 42	3963 ± 42	**311 ± 29 (p < 0.001)** **− 33.5**	**1113 ± 93 (p = 0.003)** **− 29.9**	4363 ± 145 (*p* = 0.150) 10.1	**240 ± 3** **(p < 0.001)** **− 48.7**	**737 ± 48 (p < 0.001)** **− 53.6**	4361 ± 4 (*p* = 0.153) 10.0

The negative symbol in the percentage difference indicates metal artifacts reduction with the reference beam of 120 kV

For the needle artifact, the most considerable artifact reduction achieved by using the Ag filter compared to the conventional beam energies used in CT was found for FWTM (reduction of − 53.3%) concerning the beam of 120 kV. For the needle tip and trajectory artifact, the largest reduction was obtained for absolute error, with a reduction of − 58.3% and − 49.9%, respectively, concerning the beam of 100 kV.

### Image noise

The ranges (minimum to maximum) of image noise for the dataset of 100 kV, 120 kV, 135 kV, and 120 kV-Ag were 11.1–12.9, 94.3–96.1, 103.1–106.2, 11.0–12.3, and 9.5–11.8 HU for the ROI in the heart, left lung, right lung, tissue 1, and tissue 2, respectively. No significant differences were found (*p* > 0.05).

## Discussion

Reducing metal artifacts from metallic trocars in CT interventional procedures is essential to ensure a successful treatment. Most studies have typically focused on reducing artifacts in patients with metal implants, such as a hip prosthesis placed in a fixed position [16, 17]. However, few methods have been proposed to reduce metal artifacts generated during CT interventional procedures. This study explores the metal artifact reduction capabilities of a method based on beam hardening by incorporating a 0.5 mm Ag additional filter in an MDCT. The presented results highlight a new form of metal artifact reduction when the 120 kV-Ag filter was used. For needle tip and trajectory artifacts, a significant reduction of − 53.6% (*p* < 0.001) and − 48.7% (*p* < 0.001) in the drop of the HU was found, respectively, in comparison with the reference beam of 120 kV, as well as a significant decrease of up to − 34.7% in the artifact width (width at − 100 HU, *p* < 0.001). In addition, a significant reduction in the blooming artifact of − 14.2% (FWHM, *p* < 0.001) and − 53.3% (FWTM, *p* < 0.001) was found in the needle artifact.

Because of the continuous change of trocars position within the patient and the need to obtain images in real-time, have been challenging to develop methods for reducing metal artifacts caused for a needle biopsy. Currently, we are not aware of any vendor allowing their metal artifact reduction algorithms to be applied to real time interventional CT data because of timeliness requirements for image presentation. High tube voltage, increased tube current, narrow collimations, greater slice thickness, and extended CT scale have not significantly improved artifact reduction and could increase the patient’s dose [18]. Iterative metal artifact reduction (iMAR) algorithms have shown important metal artifact reduction from the biopsy needles and antennas for microwave ablation, especially regarding photon starvation artifacts. However, the generation of new artifacts with additional blooming artifacts around the trocars and peripherical dark streaks has also been observed [3, 19]. Overall, based on beam hardening and using an Ag additional filter, the method presented in this work significantly decreased the severity of metal artifacts, improved structures’ visibility, and improved target structures’ correct location without changing the image quality.

FWHM and FWTM values, measured on the needle artifact, represent the degree of the blurring effect in the needle, which should be low enough not to change the actual size of the needle on the CT image. The CT often overestimates the needle width due to blooming artifacts [3, 19–21]. In this work, the smallest FWHM and FWTM values were obtained for the beam of 120 kV-Ag, which means a value closer to the actual diameter of the needle and less blooming artifact in the CT image. Differences between FWHM values for the 120 kV-Ag filter and the reference beam of 120 kV with the actual needle gauge of 1.27 mm were 0.61 mm (48%, 120 kV-Ag) vs. 0.92 mm (72%, 120 kV).

Changes in the width of the needle tip and trajectory artifacts for each beam energy were assessed with the FWHM and FWTM values obtained from profiles placed in the dark region after the needle tip. The minor artifact widths were obtained for the 120 kV-Ag for all cases. However, significant differences with the reference beam of 120 kV were obtained only for the needle tip artifact, with reductions of − 14.8% (FWHM, *p* < 0.05) and − 23.2% (FWTM, p < 0.05). No significant changes to the trajectory artifact could be explained because there is also an increase in profile height (larger decrease in HU) when the width increases, which causes the FWHM and FWTM values not to show such significant changes. An alternative metric to compare the artifact width considering the artifact changes in the profile height, is to measure the width for a constant value. This work measured the width for a constant value of − 100 HU in the trajectory artifacts. A significant reduction of − 34.7% (*p* < 0.001) was obtained when the 120 kV-Ag filter was used compared with the reference beam of 120 kV. This metric becomes vital since a physician would not typically re-window and level the image to optimize the visualization of the artifact region, so what is a good metric for the artifact’s negative effect is the artifact’s width at a set window width and level.

Measures of the absolute error on the needle tip and trajectory artifacts represent the maximum darkening in the hypodense areas commonly observed extending from the needle tip. A small absolute error value indicates a minor change in the HU caused by the artifact. This study found the smallest absolute error values for 120 kV-Ag, which indicates that it generates minor hypodense regions. The absolute error for 120 kV-Ag compared with the 120 kV reference beam showed a significative reduction of − 48.7% (reduction of 228 ± 8 HU, p < 0.001) and − 53.6% (reduction of 850 ± 63 HU, p < 0.001) for needle tip and trajectory artifacts, respectively.

During the reconstruction process, the image noise is magnified in the artifact’s areas and could affect other adjacent regions [22]. Besides, it is well known that increased energy may affect image contrast due to a higher proportion of Compton interactions and more forward scatter reaching the image receptor [23, 24]. This work assessed the image noise in five regions outside the artifact area and found no significant change (*p* > 0.05 Friedman test), considering the phantom dose was kept constant in all beam energies. This result shows that the Ag filter can reduce needle artifacts while maintaining image quality and performance. An additional benefit of using the Ag filter for some studies, such as pulmonary, could be the radiation dose reduction to the patient. Nomura et al. [25] showed that the Ag filter decreased the patient dose while maintaining image quality in CT localizer radiography.

This study has several limitations. We evaluated the artifact reduction only for an adult torso phantom. However, evaluations with patients should be investigated. Using the Ag additional filter for larger patients is expected to improve the x-ray detection efficiency owing to x-ray beam hardening. Therefore, large patients could benefit more from this method than smaller patients. Nevertheless, for smaller patients use of the 120 kV-Ag will likely decrease soft tissue and iodine contrast, but that isn’t usually a limitation in the setting of interventional CT where the imaging tasks are inherently high contrast. Further evaluation of tissue contrast, noise, and artifacts should be performed as a function of patient size. Furthermore, we did not evaluate the artifact reduction and image quality, considering other parameters that could affect the artifact degree and image noise, such as the needle gauge.

## Conclusion

A 0.5 mm Ag additional MDCT filter demonstrated consistent metal artifact reduction generated by the biopsy needle. This reduction may lead to an increase in image quality and a better depiction of the target and surrounding structures allowing for better needle placement during CT-guided interventional procedures.

## Data Availability

Data is provided within the manuscript.

## Abbreviations

Ag: Silver
MDCT: Multidetector Computed Tomography
CT: Computed Tomography
HU: Hounsfield Unit
AE: Absolute Error
FWHM: Full width at half maximum
FWTM: Full width at one-tenth of maximum
